# Bioecological representations and social characteristics of students influence their attitudes toward wild vertebrates

**DOI:** 10.1186/s13002-023-00593-5

**Published:** 2023-06-12

**Authors:** Amanda Rozendo da Silva, Franciany Braga-Pereira, Anna Karolina Martins Borges, José Valberto de Oliveira, Moacyr Xavier Gomes da Silva, Rômulo Romeu Nóbrega Alves

**Affiliations:** 1 Laboratório de Etnobiologia, Universidade Estadual da Paraíba, Avenida das Baraúnas, 351, Bairro Universitário, Campina Grande, PB 58429-500 Brazil; 2 Departamento de Sistemática e Ecologia, Universidade Federal da Paraíba, João Pessoa, PB 58051-900 Brazil; 3 Programa de Pós-Graduação em Etnobiologia e Conservação da Natureza, Universidade Federal Rural de Pernambuco, Av. Dom Manoel de Medeiros, s/n – Dois irmãos, Recife, PE 52171-900 Brazil

**Keywords:** Ethnozoology, Wildlife, Empathy, Antipathy, Conservation

## Abstract

**Background:**

The origin of different human emotions directed towards animals (whether in the utilitarian, affective, conflictual, or cosmological context) is strongly influenced by sociocultural factors, although our genetic predispositions also play an important role in the origin of these emotions. Such emotions guide people’s representations of different species, which in turn affect their attitudes toward them. For this reason, understanding the factors that guide such attitudes becomes a key element in making conservationist decisions. In this sense, the main objective of this study was to analyze how sociocultural characteristics and bioecological representations can influence students’ attitudes of empathy or antipathy towards vertebrate species; as well as which classes and species are related to greater and lesser support in people for their conservation.

**Methods:**

To do so, 667 interviews were conducted with students from urban (n = 1) and rural (n = 2) schools in the Brazilian semi-arid region. We used mixed generalized linear models (GLMM) to examine the effect of social factors and bioecological representations on empathy and antipathy attitudes and multiple factor analysis (MFA) to examine the relationship between the biological characteristics of the animals (positive or negative) and the attitudes toward them (antipathetic or empathetic).

**Results:**

Through GLMM, we found that students from the urban area and from lower school levels are more extreme in their responses, more frequently expressing both empathy and antipathy towards wild animals. Regarding gender, women had a higher frequency of responses associated with aversion than men for species perceived as dangerous and poisonous (*p* < 0.001). Through the MFA, we found greater support (empathy) for the conservation of fish species (31.56%), birds (29.37%) and mammals (25.94%), with emphasis on the Red-cowled cardinal (*Paroaria dominicana*) and clownfish (*Amphiprion ocellaris*) species, and less support (antipathy) for reptile and amphibian species such as rattlesnakes (*Crotalus durissus*) and horned frogs (*Ceratophrys joazeirensis*).

**Conclusions:**

The attitudinal ambivalence reflected by varying empathy for certain species and antipathy to others has important implications for wildlife conservation. Understanding the socioeconomic factors and emotions that influence attitudes towards animals can enable integrating educational strategies for the conservation of species, especially those which are culturally important.

**Supplementary Information:**

The online version contains supplementary material available at 10.1186/s13002-023-00593-5.

## Background

The coexistence between human societies and wild animals is manifested in utilitarian contexts (uses as food, transport, clothing, and various raw materials); affective (arousing admiration and sympathy) [1], cosmological [2], conflictual (species that can cause real or potential harm to people) [3–6] among others. These various interaction forms guide the representations and attitudes of humans toward other animal species, reflecting on relationships between people and animals that can be harmonious or not. In this context, Wilson [7] proposed the theory of Biophilia, which refers to an innate and positive human predisposition of affiliation to the natural environment, which allows the human being to experience benefits that, according to its author, facilitated the development, adaptation, and survival of human beings. On the other hand, the biophobic component of connectedness with nature has been registered by different authors, who describe it as the feeling of fear or rejection of natural elements with an adaptive purpose [8, 9].

Biological representations, as explained by the Bronfenbrenner theory [10, 11], can also influence human attitudes towards animals, as an individual’s genotype and biological characteristics can shape their perceptions and behaviors towards animals within their immediate environment. From an evolutionary perspective, the emergence of emotions in relation to animals would be linked to adaptation and problem-solving factors in different environments [12]. Human populations from different regions of the globe have similar biological predispositions, which makes them have similar emotions towards animals [13, 14]. For example, the fear of pointed-shaped structures (e.g., teeth, claws, animals with zigzag skin) corresponds to an emotion that evolved to solve adaptive problems, enabling human species all over the world to avoid threats to their survival [15, 16].

On the other hand, the coexistence between humans and other animal species is also influenced by sociocultural and genetic characteristics particular to each person, thereby resulting in a diverse range of emotions related to animals [7, 17–20]. Sociodemographic factors can enhance the charisma directed to animal species. All these emotions in relation to wild fauna guide human perceptions and attitudes, including those which may or may not support the conservation of certain species [21]. The wide range of perceptions with a greater degree of antipathy or empathy of people regarding different vertebrate species influences human attitudes toward fauna [22, 23].

Studies have identified a greater affinity and support for protection by people in relation to aesthetically more attractive, utilitarian, and sometimes charismatic species, such as some species of birds, mammals, and fish [24–26]. Conversely, there is a tendency to dislike animals that are considered ugly or perceived as harmful, such as bats, spiders, amphibians, and reptiles [27–29]. Therefore, it becomes important to highlight the ecological role of species considered dangerous or disgusting as a way to mitigate negative attitudes towards them [12]. Thus, understanding the underlying criteria that influence preferences may reveal useful information for the development of conservation strategies. Among the strategies, formal education plays a key role in contributing to the reconstruction of knowledge [30] and, consequently, of perception and changes in behaviour [31–34]. In fact, some studies showed that the public positive attitude toward controversial wildlife species, such as sharks, wolves, and alligators, can be improved with conservation education programs [35–37].

In this study, we analyzed how socioeconomic characteristics and bioecological representations can influence the attitudes of empathy or antipathy of elementary school students towards 25 species of vertebrates (from different classes). We expect that there will be variation in students’ attitudes according to vertebrate taxa, with empathy mainly associated with species phylogenetically closer to humans, perceived as useful, as well as considered important for nature. We also expect that students’ attitudes towards taxa are influenced by socioeconomic factors such as school location (rural and urban), education level, their gender and age.

## Methods

### Study area

**Fig. 1:**
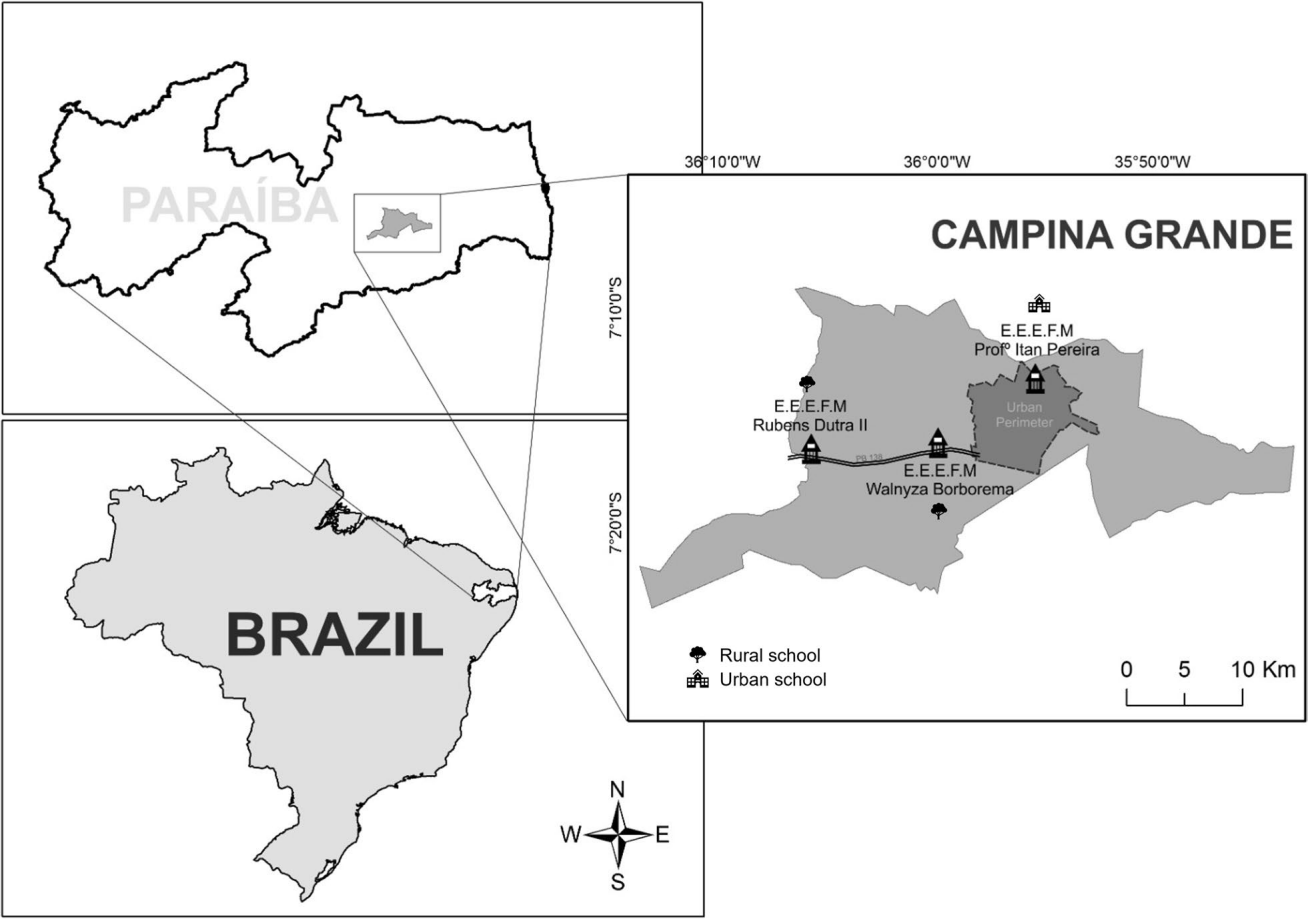
Map showing the location of the schools where the research was carried out, Municipality of Campina Grande, PB, Brazil

First, three schools were selected to obtain the data, with one being urban and two rural in the State Network, all located in the Municipality of Campina Grande, PB (07° 13′ 50″ S 35° 52′ 52″ W), in Northeast Brazil (Fig. 1). We used school units that included Elementary School (6th to 9th grades). Two rural schools were inserted so that the urban and rural sample sizes were approximately the same, since the number of students in rural schools is smaller. The schools chosen for the study were the following: (1) Itam Pereira, State Elementary and Secondary School, located in the western urban zone of the municipality, created by Decree no. 21,039/2000; (2) State Elementary and Secondary School Rubens Dutra Segundo, located in the District of Catolé de Boa Vista, 26 km west of the center of the municipal seat with access via BR 230, and created by Decree No. 13151/1989; and (3) State Elementary and Secondary School Walnyza Borborema Cunha Lima, located in Sítio Estreito, 12 km west of the center of the municipal seat, with access via BR 230, and created by Resolution 36.730/2006/2016 (Fig. 1).

### Ethical aspects of the study

The study was carried out in accordance with the requirements of ethical/legal procedures, being approved by the Research Ethics Committee of the State University of Paraíba (Protocol CEP-UEPB: 43589815.0.0000.5187). The data collection was possible due to a sequence of institutional requirements. First, we obtained authorization from the major educational instance in the state and, subsequently, from each school director. As the questionnaires would be applied during Science/Biology classes, we obtained authorization from the respective teachers, who also participated in the data collection as facilitators. In compliance with the Committee’s requirements, we sent the Informed Consent Forms (ICF) and an ethical/legal requirement for effective participation in the research process to the parents and/or guardians of the interviewed students. More than 90% of the parents authorized their children to participate in the study. The students also received an explanation about the research and its objectives, the questions they should respond to, and their rights (e.g., anonymity, withdrawing the study at any point). For that, we count on the help of the teachers, to make the communication most understandable as possible and to establish trust. After that, all the students agreed to participate in the study.

### Data collection

Data collection took place from June to December 2015, through semi-structured questionnaires applied during 38 classes of science and biology in Elementary School. A total of 667 students participated in the survey, 383 urban and 284 rural, aged between 9 and 17 years old, 334 men, and 333 women.

Students were shown boards containing images of 25 species distributed among taxa: fish, amphibians, reptiles, birds, and mammals (Table 1). The species shown (1 at a time) were randomly exposed to the students using an overhead projector (see Fig. 1 in Additional file 1: Questionnaire S1). There was no additional information about these species, and the students were monitored during the application of the questionnaire so that there was no interference between them in the answers.

**Table 1:** Species projected to students in the study, with the respective conservation status and reference on what the selection was based on

Scientific name	Common name	Conservation status (IUCN)	References
Mammals			
* Artibeus lituratus* (Olfers, 1818)	Great fruit-eating bat	LC	[38, 39]
* Callithrix jacchus* (Linnaeus, 1758)	Common mamorset	LC	[40]
* Cavia aperea* (Erxleben, 1777)	Brazilian guinea pig	LC	[41]
* Euphractus sexcinctus* (Linnaeus, 1758)	Six-banded armadillo	LC	[40]
* Panthera onca* (Linnaeus, 1758)	Jaguar	NT	[39]
Birds			
* Caracara plancus* (Miller, 1777)	Southern caracara	LC	[42]
* Coragyps atratus (*Bechstein, 1793)	American black vulture	LC	[43]
* Glaucidium brasilianum* (Gmelin, 1788)	Ferruginous pygmy-owl	LC	[43]
* Paroaria dominicana* (Linnaeus, 1758)	Red-cowled cardinal	LC	[44]
* Patagioenas picazuro* (Temminck, 1813)	Picazuro pigeon	LC	[43]
Reptiles			
* Caiman crocodilus* (Linnaeus, 1758)	Spectacled caiman	LC	[45]
* Chelonoidis carbonarius* (Spix, 1824)	Red-footed tortoise	NE	[46]
* Crotalus durissus* (Linnaeus, 1758)	Cascabel rattlesnake	LC	[47, 46]
* Iguana iguana* (Linnaeus, 1758)	Common green iguana	LC	[46]
* Salvator merianae* (Duméril & Bibron, 1839)	Black-and-white tegu	LC	[22, 23, 46]
Amphibians			
* Ambystoma maculatum* (Shaw, 1802)	Spotted salamander	LC	[48]
* Ceratophrys joazeirensis* (Mercadal de Barrio, 1986)	Caatinga horned frog	LC	[49]
* Leptodactylus vastus* (Lutz, 1930)	Northeastern pepper frog	LC	[50]
* Pithecopus nordestinus* (Caramaschi, 2006)	Tree frog	DD	[51]
* Rhinella jimi* (Stevaux, 2002)	Jimi toad	LC	[52]
Fishes			
* Amphiprion ocellaris* (Cuvier, 1830)	Clown Anemonefish	LC	[53]
* Galeocerdo cuvier* (Péron & Lesue, 1822)	Tiger shark	NT	[54]
* Diodon hystrix* (Linnaeus, 1758)	Spot-fin Porcupinefish	LC	[55]
* Hippocampus reidi* (Ginsburg, 1933)	Longsnout seahorse	NT	[55]
* Hoplias malabaricus* (Bloch, 1794)	Trahira	LC	[17]

Conservation status based on IUCN Red List of Threatened Species (https://www.iucnredlist.org/). LC = Least Concern; NT = Near Threatened; DD = Data Deficient; NE = Not Evaluated

As a criterion for choosing vertebrates, we considered species that occur in the study region based on ethnozoological studies carried out in the region and in Brazil, as well as exotic species, including animals considered “charismatic” or with utilitarian value for humans, and others that are “conflict targets” or that are related antipathy for being historically stigmatized according to consulted literature (Table 1).

After the projection with the image of each species, the research participants were asked to answer a total of 22 sentences of representations and attitudes in relation to each species (see Additional file 1: Questionnaire S1). Each sentence contained a statement, and the student should indicate how much they agreed with a such statement within a Likert scale ranging from 0 to 10 [56]; the closer to 10, the greater the agreement with the proposed sentence. We divided the sentences related to the representations into: (1) positive bioecological representations and (2) negative bioecological representations; and those related to an attitude in: (1) attitudes of empathy; (2) attitudes of antipathy; and (3) attitudes of antipathy (Table 2). The questions referring to the socioeconomic information of the students involved in the study were included in the same questionnaire. The average time to perform each board was about 25 min.

**Table 2:** Set of bioecological representations and attitudes of empathy and antipathy

*Positive bioecological representations*
It’s a useful animal
It is a completely harmless animal
It usually ignores humans
It usually runs away from humans
It is important for nature
*Negative bioecological representations*
It’s a dangerous animal
It’s a poisonous animal
It’s a fatal animal for humans
It tends to attack humans
*Empathy attitudes*
I like the animal
I like being close to this animal
I don’t care if the animal lives in my house/property
I agree that this animal is protected by law
*Antipathy attitudes*
I think the animal is ugly
I don’t go close to it
I don’t like the noise the animal makes
I’m afraid of the animal
*Extreme antipathy attitudes*
I can’t stand this animal
The animal gives me nightmares
The animal should be extinct
If there was a population of this animal in my yard or property I would take steps to eliminate it
I usually kill it when I find it or ask someone for help to kill it

### Data compilation

Considering the Likert scale score indicated by each student to answer each sentence, the average value of the scores of the sentences that form each set of representations and attitudes mentioned above was calculated by student and species.

### Data analysis

We used mixed generalized linear models (GLMM) with negative binomial distribution to examine the degree of effect of social factors and bioecological representations on empathy and antipathy attitudes. We used GLMM because we transformed the ordinal values obtained for each attitudinal sentence into continuous values when calculating the average of the scores obtained in each sentence of the same attitude set. We considered the student as a predictor variable of random effect (control), while social factors and bioecological representations were considered predictor variables of fixed effect. We tested the collinearity (*p* > 0.05) between the predictor variables prior to the analyses. We performed residual analysis to check whether or not our models were suitable in principle. Using the Akaike information criterion, the models were selected considering ΔAIC values > 6 when calculating the difference in the AIC value of the null model in relation to the AIC value of the selected model (the one that included all the uncorrelated predictor variables of interest). All analyzes were performed in R ver. 3.5.3 [57] using the MuMin and lme4 packages [58, 59]*.*

Next, we performed a multifactor analysis (MFA) to verify whether taxa (species or class) that are perceived as having negative bioecological characteristics (such as dangerous and useless) are more frequently associated with antipathetic attitudes; and if taxa that are perceived as having positive bioecological characteristics (such as useful to people and important to nature) are more frequently associated with empathetic attitudes. Compared to the frequently used principal component analysis (PCA), MFA takes into account that the data are structured in sets [60, 61] (herein different sentences, and that each student is considered as a sampling unit, as well as the species or their classes), depending on the data analyzed. Each species (or class) then becomes more important within a given set of sentences when it receives higher scores repeatedly by several students. The MFAs were based on the FactoMineR package [62] for the analyzes, and Factoextra [63] for data visualization.

## Results

### Effect of socioeconomic variables on empathy and antipathy attitudes

Regarding the school locations, our results indicate that students from the urban area responded with higher scores to most sentences for both those regarding empathy and antipathy attitudes towards wild animals compared to students from the rural area, who are less extremist in their responses (*p* < 0.05). Students at lower school levels also have more extreme responses, agreeing more frequently with empathy (*p* < 0.001) and extreme antipathy (*p* < 0.05) attitudes when compared to students at higher school levels. Moreover, women showed greater aversion to animals, significantly scoring antipathy and extreme antipathy attitudes (Table 3).

**Table 3:** Effect of different socioeconomic representations on the attitude of empathy, antipathy, and extreme antipathy of students towards wild animals

Response variables	Predictor variables	Estimate	Std.Error	z value	Pr( >|z|)		AIC	AIC Null model	ΔAIC
Empathy attitudes							71,515	73,352	1837
	Urban: rural	0.169253	0.072158	2.346	0.018997	*			
	Grade	− 0.04893	0.014144	− 3.459	0.000541	***			
	Age	0.010985	0.008285	1.326	0.184833				
	Male:Female	− 0.0068	0.023222	− 0.293	0.769584				
	Family income	0.011157	0.011729	0.951	0.34148				
Antipathy attitudes	Urban: rural	0.152115	0.076199	1.996	0.0459	*	67,891.4	9882.9	1991.5
	Grade	− 0.02099	0.014952	− 1.404	0.1604				
	Age	− 0.00089	0.008744	− 0.102	0.9189				
	Male:Female	− 0.25815	0.024605	− 10.492	< 2e16	***			
	Family income	0.01432	0.012416	1.153	0.2488				
Extreme antipathy attitudes							56,706.9	58,328.5	1621.6
	Urban: rural	0.106432	0.153852	0.692	0.4891				
	Grade	− 0.07491	0.029836	− 2.511	0.0121	*			
	Age	0.01222	0.017345	0.705	0.4811				
	Male:Female	− 0.22569	0.04889	− 4.616	3.91E-06	***			
	Family income	0.031917	0.024814	1.286	0.1983				

Estimated values indicate the coefficient associated with the variable listed on the left. This represents the estimated amount by which the odds (log x) of each response variable would increase if each explanatory variable were one more unit. Standard errors are an average estimate of how much any response variable would fluctuate if the study were run again identically, but with new data. Z values indicate the degree to which the explanatory variables have a significant effect. Pr ( >|z|) are listed as two-tailed p-values that correspond to z-values following a standard normal distribution. Significance levels as follows: *P* > 0.05; *P ≤ 0.05; ***P ≤ 0.001

### Effect of bioecological representations on students’ attitudes

**Fig. 2:**
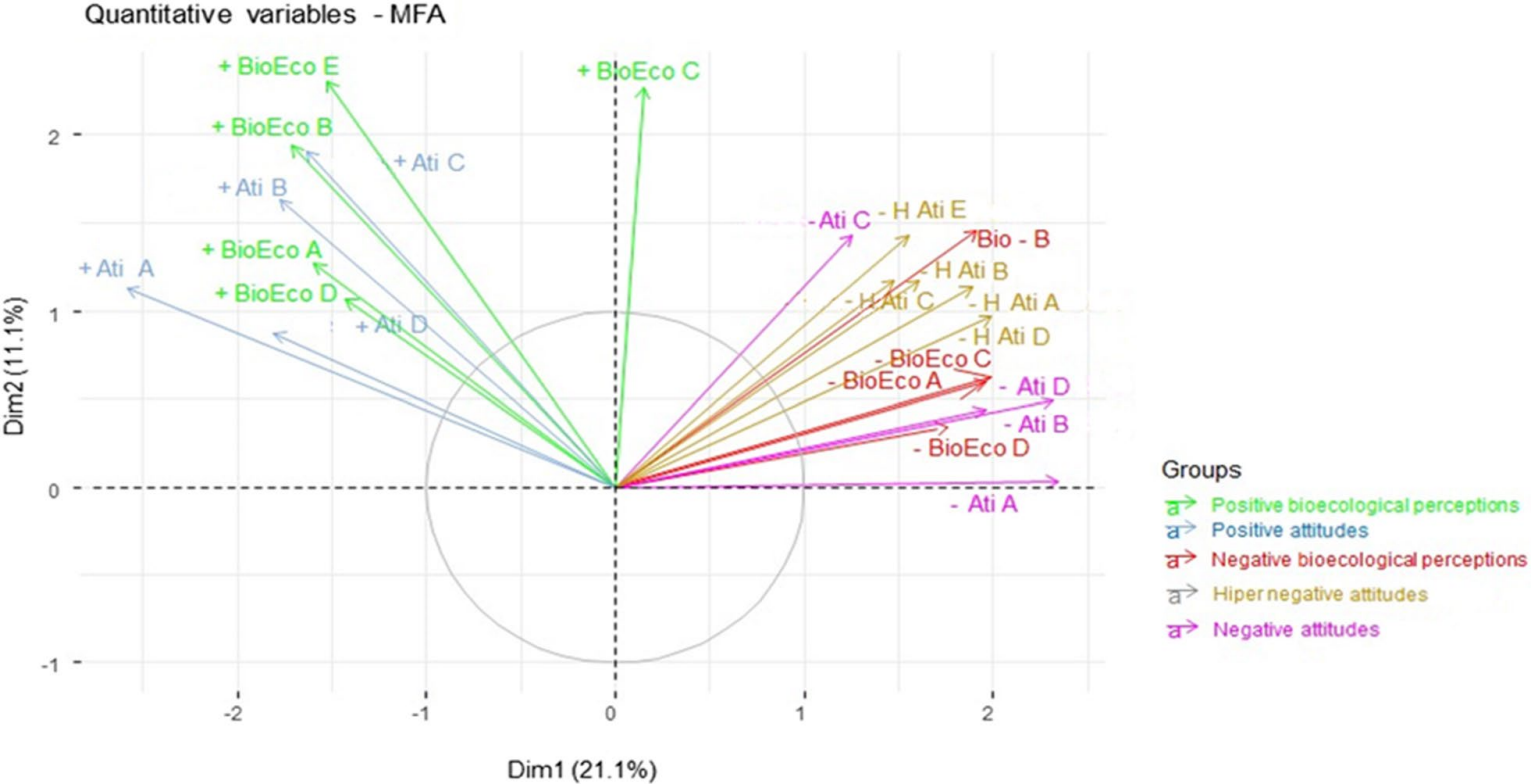
MFA result for variable sets. Positive bioecological representations (green) (+ BioEc A = useful; + BioEco B = harmless; + BioEo C = ignores humans; + BioEco D = runs away from humans and + BioEcoE = important for nature). Positive attitudes (blue) (+ Ati A = I like the animal; + Ati B = I like being close to it; + Ati C = I don’t care if the animal lives in my house; + Ati D = I agree that this animal is protected by law). Negative bioecological representations (red) (-BioEco A = dangerous; -BioEco B = poisonous; -BioEco C = fatal; -BioEco C = attacks humans. Negative attitudes (violet) (–Ati A = ugly animal; -Ati B = I don’t go near it; –Ati C = I don’t like the noise; –Ati D = I’m afraid) Extreme negative attitudes (yellow) (–H Ati A = I can’t stand the animal; –H Ati B = the animal gives me nightmares;–H Ati C = it should be extinct; HAti D = If there was a population of this animal on my property I would take measures to eliminate it) and HAti E = I usually kill it when I find it or ask someone for help to kill it)

We found greater agreement in the statements of empathetic attitudes towards species that are perceived as useful, harmless and important to nature (*p* < 0.001), as well as greater disagreement in the antipathy and extreme antipathy attitudes towards species that are considered important to nature, harmless and useful for people. On the other hand, we found agreement with antipathy and extreme antipathy attitudes towards those species perceived as dangerous, poisonous, fatal, which attack or that ignore people (*p* < 0.001) (Table 4, Fig. 2).

**Table 4:** Effect of different bioecological representations (positive and negative) on the attitude of empathy, antipathy, and extreme antipathy of students towards wild animals

Response variables	Predictor variables	Estimate	Std. Error	z value	Pr( >|z|)		AIC	AIC Null model	ΔAIC
Empathy attitudes	Useful	0.038687	0.00294	12.94	< 2.00E16	***	68,718. 3	73,416	4697.7
	Harmless	0.029232	0.00259	11.26	< 2.00E16	***			
	Ignores humans	0.008717	0.00268	3.292	0.00095	***			
	Runs away from humans	0.01636	0.00254	6.38	1.77E-10	***			
	Important for nature	0.047723	0.00277	17.39	< 2.00E16	***			
	Dangerous	− 0.02545	0.00344	− 7.304	2.80E-13	***			
	Poisonous	− 0.0308	0.00299	10.32	< 2.00E16	***			
	Deadly	0.009433	0.00353	2.662	0.00779	**			
	Attacks humans	0.016471	0.00332	4.958	7.13E-07	***			
Antipathy attitudes	Useful	− 0.02428	0.00326	− 7.481	7.38E-14	***	65,503.9	69,874.4	4370.5
	Harmless	− 0.01774	0.002774	− 6.395	1.61E-10	***			
	Ignores humans	0.013108	0.002838	4.619	3.85E-06	***			
	Runs away from humans	0.007412	0.002637	2.811	0.00494	**			
	Important for nature	− 0.02069	0.002947	− 7.02	2.21E-12	***			
	Dangerous	0.028819	0.003385	8.514	< 2.00E16	***			
	Poisonous	0.030866	0.00302	10.22	< 2.00E16	***			
	Deadly	0.02334	0.003474	6.719	1.83E-11	***			
	Attacks humans	0.033128	0.003274	10.118	< 2.00E-16	***			
Extreme antipathy attitudes	Useful	− 0.04337	0.003743	11.588	< 2.00E-16	***	53,403.6	58,326.2	4922.6
	Harmless	− 0.00748	0.003054	− 2.448	0.014364	*			
	Ignores humans	0.029906	0.003185	9.39	< 2.00E-16	***			
	Runs away from humans	− 0.01277	0.002932	− 4.355	1.33E-05	***			
	Important for nature	− 0.01153	0.003325	− 3.467	0.000527	***			
	Dangerous	0.036871	0.003834	9.618	< 2.00E-16	***			
	Poisonous	0.088817	0.003248	27.347	< 2.00E-16	***			
	Deadly	0.009856	0.003912	2.519	0.01176	*			
	Attacks humans	0.01134	0.003699	3.066	0.002173	**			

Estimated values indicate the coefficient associated with the variable listed on the left

Z values indicate the degree to which the explanatory variables have a significant effect. Pr ( >|z|) are listed as two-tailed p-values that correspond to z-values following a standard normal distribution. Significance levels as follows: *P* > 0.05; *P ≤ 0.05; **P ≤ 0.01; ***P ≤ 0.001. Quantitative statistics for the score obtained through the Likert scale for empathy and antipathy attitudes. Estimated values, standard error, z values and significance as predicted by the GLM model

### Effect of variables of representations and attitudes according to taxa

**Fig. 3:**
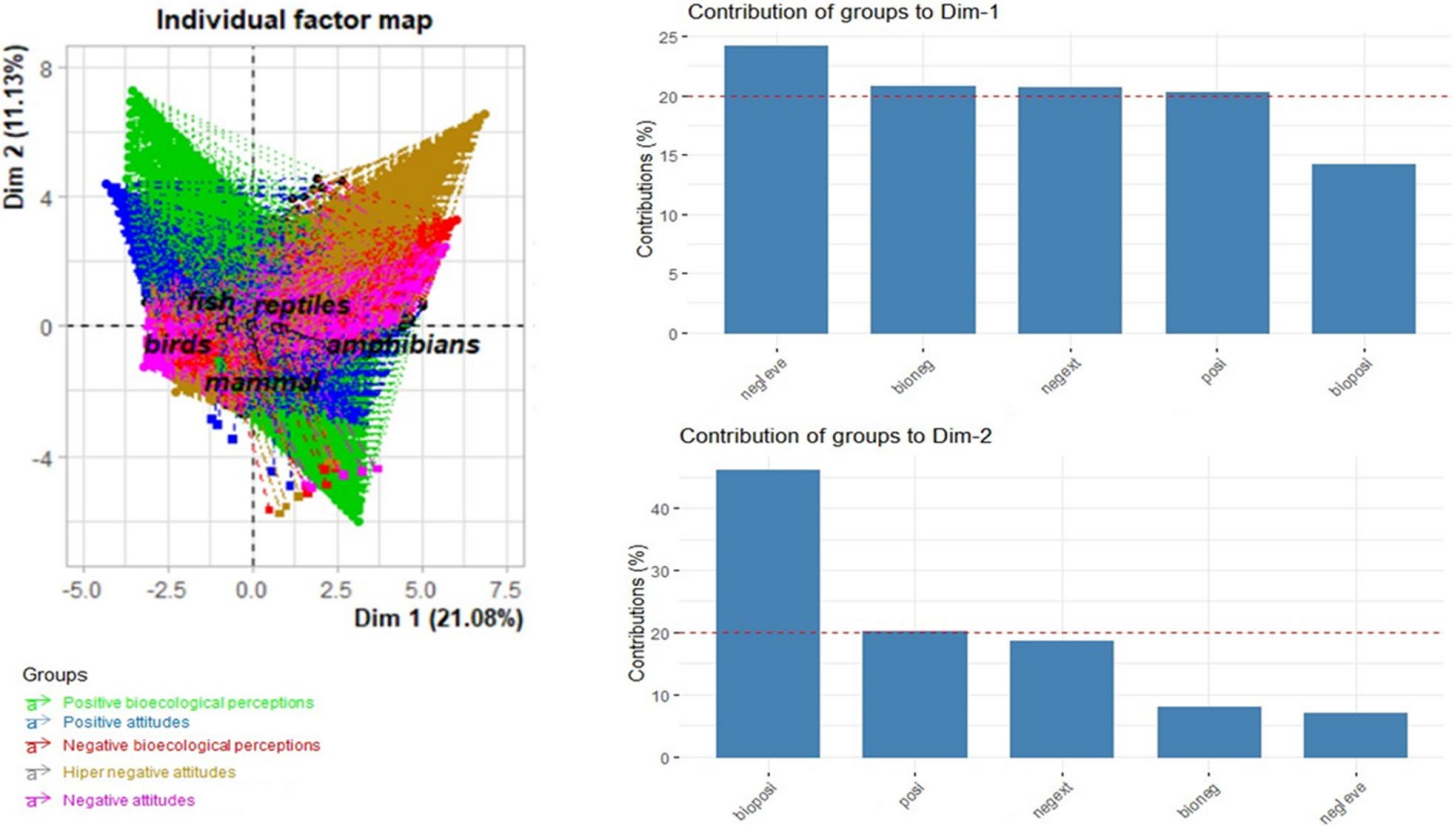
Effect on variations in representations and attitudes according to student taxa from a multifactor analysis (MFA)

**Fig. 4:**
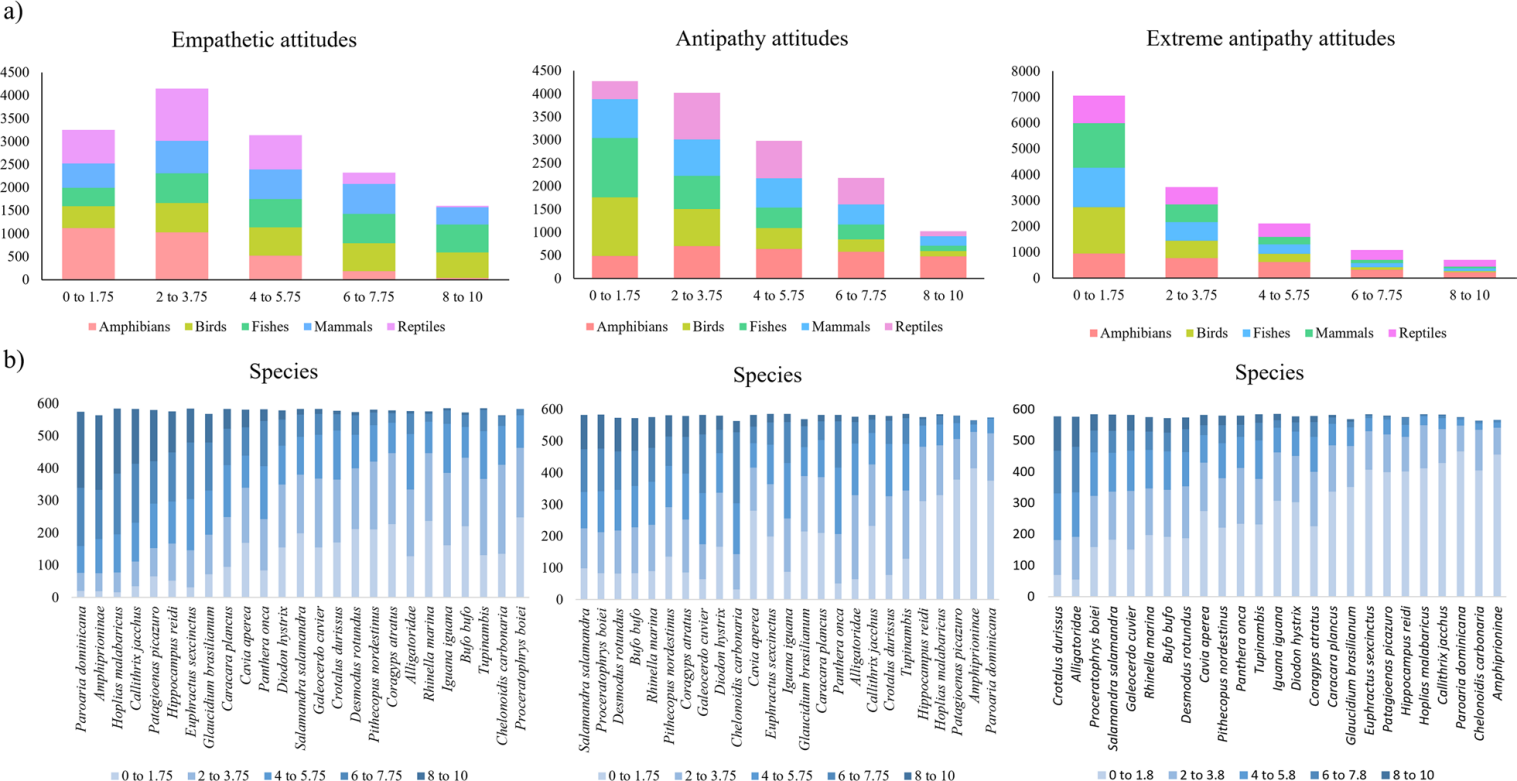
Frequency of agreement with sentences of empathy, antipathy and, extreme antipathy for each taxon. The x-axis is formed by the frequency of the number of samples and attitudes answered proportionally to the number of students who cited each score for each species; and the y axis is formed by the scores of the sets of attitudes. These values were scored to form five scores based on a degree of agreement: (0 to 1.75 = strongly disagree), (2 to 3.75 = strongly disagree), (4 to 5.75 = disagree), (6 to 7.75 = agree) and (8 to 10 = I totally agree)

We found a high frequency of positive representations and empathetic attitudes mainly directed towards fish (31.56%), birds (29.37%) and mammals (25.94%) (Fig. 3). When observing each isolated sentence, we found that the sentences “I like the animal”', “I don’t care if the animal lives in my house”' or “I agree that the animal is protected by law” were more observed for birds and fish in relation to mammals (Fig. 4A).

On the other hand, we found a high frequency of negative representations and attitudes of antipathy towards reptiles and amphibians (Fig. 3). More specifically, when comparing each sentence itself, we found that there was high agreement with the sentences “ugly animal”, “I'm not going near” or “I'm afraid” for amphibians, which denote attitudes of antipathy. We found high agreement with the sentences “I can’t stand the animal”, “the animal gives me nightmares” or “this animal should be extinct” for reptiles, which denote attitudes of extreme antipathy (Fig. 4A).

Amphibians and reptiles are the most representative taxa in the first dimension (Dim 1 = 21.08%) composed of negative bioecological representations that influence negative attitudes of antipathy (whether these are extreme or not). Meanwhile, the group formed by taxa of fish and birds (Dim 2) explains 11.13% of the positive bioecological representations that justify empathy attitudes (Fig. 3).

The species which stood out with the greatest consensus of empathy among fish was the “clownfish” (*A. ocellaris*), an exotic animal in Brazil. The “wolf-fish” (*H. malabaricus*) and the “longsnout seahorse” (*H. reidi*) also scored empathetic attitudes, however the “tiger shark” (*C. cuvier*) was the species which presented less empathy. Regarding birds, the species with the highest empathy consensus were “red-cowled cardinal” (*P. dominicana*) and “Picazuro pigeon” (*P. picazuro*) native to the study region. On the other hand, less empathy was observed for the “black vulture” (*C. atratus*). The species with the highest empathy consensus among mammals were the “marmoset” (*C. jacchu*), “six-banded armadillo” (*E. sexcinctus*) and the “jaguar|” (*P. onca*), while less empathy was observed for the “the great fruit-eating bat” (*A. lituratus*) (Fig. 4B).

Those with the highest antipathy scores at the species level were the “spotted salamander” (*A. maculatum*), horned frog (*C. joazeirensis*) and “Northeastern pepper frog” (*L. vastus*) (amphibians), and “the great fruit-eating bat” (*A. lituratus*). Species of amphibians and reptiles were the ones with the highest responses of extreme antipathy, with emphasis on rattlesnakes (*C. durissus*), spectacled caiman/alligator (*C. crocodilus*), horned frog (*C. joazeirensis*) and spotted salamander (*A. maculatum*) (Fig. 4B).

## Discussion

We found a strong relationship between positive representations and empathy attitudes according to the species, as well as negative representations and aversion attitudes. Our results showed that students’ attitudes of empathy and antipathy vary depending on the animal, following a trend found in previous studies [24, 64–66]. However, when we consider the large groups of wild vertebrates, we observe greater antipathy towards representatives of reptiles and amphibians, as well as greater emphaty with representatives of fish, birds, and mammals influenced by evolutionary, ecological, and cultural issues [67–70].

The strong negative perception and antipathy towards snakes, especially to the species *Crotalus durissus*, probably occurs because snakes is related to many myths, proverbs, and stories with a negative connotation which are transmitted orally in the semi-arid region of Brazil, where the research students reside. Many of these myths are based on biblical quotations that picture snakes in a negative light, as "villains" or "evil representations", and incite the indiscriminate slaughter of various snake species, both venomous and non-venomous [23, 34]. In addition, the group is associated with fatal snakebites, which makes people fear and dislike these animals [22, 23, 47]. Negative perceptions associated with snakes are registered in several locations around the world [65, 71], a situation which represents a serious conservation problem for the species of the group, since it encourages the indiscriminate slaughter of species [22, 23].

Behavioral attitudes that imply the conservation of species can be culturally constructed from conflicting, utilitarian, evolutionary, and morphological attributes of species [34, 64, 72]. This situation was observed in the analysis of the animal groups considered in this study. For example, our results show that reptiles such as alligators and lizards are related to strong fear or disgust in people [12, 73]. Such fear these reptiles is mainly due to cultural constructions created in reaction to the possibility of conflicts and risks of accidents that these animals cause to humans [45, 64, 74]. We can say that caimans, along with snakes, are predators which are perceived as deadly (i.e., capable of killing humans), and therefore extreme attitudes of antipathy such as eliminating or extinguishing these animals were recorded in our study. On the other hand, other reptiles, such as the tortoises, received more empathetic responses from the students, as they do not present risks such as snakes and alligators, in addition to having utilitarian value and being popular pets in Brazil, as pointed out by several ethnozoological studies [22, 23, 46].

Amphibians, like snakes, are also the target of myths and legends that make them the target of disgust and aversion by people [75, 76], which explains the high agreement with negative representations and adverse attitudes on the part of the interviewed students. In a study conducted in Argentina, Brazil and Uruguay, Deutsch et al. [77] found that the antipathy to the *Ceratophrys ornata* species (a species of the same genus as *Ceratophrys joazeirensis*, which we used herein), is strongly linked to symbolism and folklore beliefs.

Some species may additionally be considered ugly and associated with feelings of disgust or fear, as was observed in a study by Prokop et al. [78], who showed that disgust was negatively related to frog intolerance. The disgust associated with amphibians can be motivated by morphological characteristics of the species, such as their slimy appearance and the naked and wrinkled skin [79]. For some authors [80, 81], this situation can be explained by the existence of an adaptive mechanism that leads us to avoid organisms which make us disgusted as a way to prevent transmission of diseases and infections. In the case of amphibians, this adaptation may be related to substances which are toxic to vertebrates in the slimy skin of many amphibians [50, 82, 83]. Students may also have associated the color of the horned frog (*C. joazeirensis*) with the presence of poison and danger, also contributing to the registered antipathy. A study carried out with Slovak students showed a significant correlation between disgust and danger in relation to the animals’ colors [84].

Fish, birds, and mammals aroused greater empathy in the students. These groups generally arouse more emphaty because they are more socially accepted than reptiles and amphibians, which is also a trend observed in other studies [1, 24, 25, 29, 70]. The clownfish (*A. ocellaris*) is a species of exotic fish in Brazil which received the most empathy from the students. Unlike amphibians and reptiles, colorful species in the case of fish can trigger positive emotions on the part of people due to the practice of keeping species with more striking colors in aquariums [53], in addition to being widely publicized as emblematic species by the media [85]. These factors may explain the empathy for the fish that gained much notoriety after the film “Finding Nemo”. The wolf-fish (*H. malabaricus*) and the longsnout seahorse (*H. reidi*) also aroused positive attitudes in students, which may have an influence on the utilitarian value of these species which are used in food or in local folk medicine, as well as pets [86, 87].

Empathy and antipathy attitudes suffer variations according to taxa. For example, sharks are related to attitudes of antipathy as opposed to the other fish mentioned. The morphological characteristics of sharks, with prominent teeth and large size, arouse an instinctive fear in most people. However, this aversion to these fish is extremely potentiated by negative information present in movies and news [88, 89].

Regarding wild birds, some have attractive coloring that motivate “positive” feelings in humans, however such attraction related to birds may represent pressure for these populations by promoting illegal trade. As an example, the red-cowled cardinal (*Paroaria dominicana*) is very charismatic and very popular as a pet in the region of the present study [43]. On the other hand, we can highlight that some species received strong signs of antipathy, such as the black vulture (*C. atratus*), crested caracara (*Caracara plancus*) and the Ferruginous pygmy owl (*Glaucidium brasilianum*). The natural necrophagous behavior regarding *Coragyps atratus* may have influenced this aversion, while conflicting stories with local populations (such as attacking domestic animal chicks) may be the reason for the aversion for the other species [64, 75]. Many birds in the northeastern semi-arid region are associated with beliefs and superstitions, including owls, which are associated with bad omens [43]. Similarly, Mikkola and Mikkola [90], recorded that 90% of respondents in their study in Africa (Malawi) also relate owls with bad luck and death.

Mammals are vertebrates that generally arouse greater emotional emphaty in people, given the relevance of this group in conservation campaigns and in the scientific literature, or due to their strong general appeal, as they are seen as utilitarian species with a pleasant appearance, in addition to their greater phylogenetic proximity to the human species [12, 21, 91]. For example, mammals such as the six-banded armadillo (*E. sexcinctus*) and marmosets (*C. jacchus*) are popular in the Brazilian Northeast region, being used as pets and food [92], respectively, and were the animals which aroused the most empathy among the interviewed students.

However, our results show that this situation depends on the mammalian species considered. Two of the mammalian species investigated, namely the great fruit-eating bat (*A. lituratus*) and the jaguar (*P. onca*) also aroused antipathy among the interviewees. In the case of *P. onca*, this fear may be associated with the potential danger that the species can cause to humans. Although jaguar attacks on humans are rare, emotions such as fear can be induced by predators which are larger and heavier than humans (as in the case of bears, wolves, and big cats) [12]. The expressive antipathy related to the bat may be associated with the potential risk of transmitting diseases, in addition to being socially stigmatized and involved in myths and beliefs with a negative connotation [38, 93, 94]. Misinformation can be an intensifier of bat disgust when considering (for example) the hypothesis that Covid-19 originated in a spillover event of pathogens from bats and pangolins to humans, which led to an increase in negative attitudes such as rabies, disgust or fear of these animals, encouraging their eradication [12].

Our results showed that the location of the students’ residence and education influenced attitudes towards animals, which reinforces the finding of Cortés-Avizanda et al. [95], who emphasize that perceptions and attitudes about wild animals vary between people and can be determined by their sociodemographic characteristics, environmental behavior and knowledge. Students in the urban location responded with higher scores to most sentences, both those of empathy and antipathy attitudes. It's not surprising that human-animal relations are influenced by socio-cultural specificities inherent to each context [96–100]. Furthermore, informal and cultural educational processes differ between urban and rural contexts [101]. For instance, formal educational processes and media access for rural students are generally less efficient than for urban students [34].

The higher scores for most empathy and extreme dislike sentences pointed out by students with lower education levels can be explained (among other factors) by the development in rationalization of reading and interpreting the world of students with higher educational levels. Despite not being a direct result of our study, age is a factor that is highly correlated with education level and can also trigger less affective emotional and more rational responses from students towards animals [64]. A study of Norwegian children and adolescents on animal-related activities showed that interest in animals decreased with increasing age [102]. In this perspective, Schlegel and Rupf [26], emphasize the different stages of child development, so that wild animals are perceived from various orientations, suggesting the following stages: affective-emotional (6 to 9 years), cognitive, knowledge-oriented (10 to 13 years old) and ethical-ecological (13 to 16 years old). Although feelings of fear and admiration for animals, which are disseminated by media vehicles, are generated at any stage of life [103], age and education are significant variables that determine the presence and intensity of emotions in relation to animals [12, 104].

Gender is among one of the factors that most influence attitudes and emotions towards animals. Other studies with both schoolchildren and adults show that women are more likely to reveal affectionate feelings towards animals, especially large and aesthetically attractive species that are considered cute and popular [26, 66, 105, 106]. Even so, we found no significant difference in responses denoting empathy between women and men. However, antipathy attitudes were more frequent among women. Women have more frequent perceptions of danger in relation to some taxa, such as amphibians, reptiles, and predatory mammals when compared to men [15, 65, 66, 78] and this may be related to biological, psychological, and cultural factors. According to the reproductive investment hypothesis, women are more concerned with protecting their children, therefore they develop greater attention to animals that present potential dangers [107]. In addition, the role assumed by men at the beginning of the evolutionary history of chasing large and dangerous animals may have influenced the greater sense of dominance over animals by males [108].

Antipathy attitudes toward wildlife are a concerning point for conservation, especially in the case of species for which human fell aversion, and we have shown here some of the drivers of these attitudes. Aesthetics have traditionally been related to societal support for species, with uncharismatic animals, such as bats, amphibians, and reptiles being targeted by persecution and receiving less directed conservation efforts as well [104, 109, 110]. Recognizing the drivers that suport positive and negative attitudes toward the animals is important to delineate effective conservation measures. Social acceptance of nature conservation actions, driven by concepts such as biophilia and positive human attitudes towards wildlife, can be a key trigger for effective conservation efforts, as it encourages individuals and communities to take ownership of conservation actions and actively participate in preserving and protecting the natural environment [111, 112].

Education can be an essential ally for change in attitudes and perceptions towards wildlife and conservation. An important strategy that can be incorporated into the school curriculum is exposure to wild animals as a way to increase knowledge and the familiarity of children with animals often considered "ugly" [67, 105]. Promoting positive attitudes towards wildlife among students is very important, and for that, the schools can also benefit from a multidisciplinary curriculum of professionals, to develop environmental education and fauna conservation projects aligned with their social and cultural context. Moreover, it is important to note that these educational efforts need to be based on continuous activities to achieve positive results in long term [113]. Furthermore, more comprehensive studies, including other socioeconomic aspects, are important to identify other drivers that can be accessed to promote change in attitudes and perceptions towards wildlife conservation and to advance human-wildlife coexistence.

## Conclusions

In conclusion, our results showed that empathy and antipathy representations and attitudes vary according to the animal considered, with amphibians and reptiles being those that receive the greatest antipathy from students. Animals considered aesthetically attractive and useful to humans tend to arouse greater empathy, while those involved in potential conflicts and risks and therefore considered dangerous or repugnant tend to more frequently related to antipathy attitudes and consequently less propensity for conservation. We have seen that both representations caused by various emotions and social factors can be key elements in making conservation decisions regarding species. Therefore, nature conservation can only be efficient if it involves the understanding of different actors in society. Children and young people have a long-term effect and are potential multipliers in the way society relates to nature. Thus, our results point to the relevance of educational strategies that foster interest and understanding in conserving wild animals.

## Supplementary Information

**Additional file 1**. Questionnaire S1.

## Data Availability

The datasets used and/or analysed during the current study are available from the corresponding author on reasonable request.
