# Supplementary material for: Bioecological representations and social characteristics of students influence their attitudes toward wild vertebrates
Source: J Ethnobiol Ethnomed. 2023 Jun 12;19:25. doi: 10.1186/s13002-023-00593-5 (PMC10258757; doi:10.1186/s13002-023-00593-5)
Supplement: Supplementary file 1 — Additional file 1. Questionnaire S1. [file 13002_2023_593_MOESM1_ESM.docx]

**Additional file 1- Questionnaire S1**

**Questionnaire applied to the students**

WILD VERTEBRATES AT SCHOOL: AN ETHNOECOLOGICAL APPROACH IN THE INTERFACE TRADITIONAL KNOWLEDGE VERSUS FORMAL BIOLOGICAL EDUCATION

SCHOOL:______________________________________________ TERM: ( ) M ( ) A

LOCATION: ( ) URBAN ( ) RURAL; GRADE/TERM:______;

CYCLE: ( ) ELEMENTARY ( ) MIDDLE

NAME:____________________________________________________________________

AGE:_____; GENDER: ( ) Male ( ) Female; RELIGION:____________________________________

FAMILY INCOME: ( ) 2 to 4 minimum monthly salaries (R$1,576.00 to 3,152.00); ( ) more than 4 minimum monthly salaries (more than R$ 3,152.00)

1. The animal shown belongs to the group: ( ) Fish; ( ) Amphibians; ( ) Reptiles; ( ) Birds;

( ) Mammals

2. What is the name of this animal? _________________________________________

3. For the animal presented, mark one option on each scale below, expressing your perception and relationship with it. The scale goes from 0 (zero) to 10 (ten); the closer to 10 the greater the intensity of your expression in relation to the proposed sentence.

| **BIOECOLOGICAL REPRESENTATIONS** | **SCALES** |
| --- | --- |
| It’s a dangerous animal | 0( ) 1( ) 2( ) 3( ) 4( ) 5( ) 6( ) 7( ) 8( ) 9( ) 10( ) |
| It’s a useful animal | 0( ) 1( ) 2( ) 3( ) 4( ) 5( ) 6( ) 7( ) 8( ) 9( ) 10( ) |
| It’s a poisonous animal | 0( ) 1( ) 2( ) 3( ) 4( ) 5( ) 6( ) 7( ) 8( ) 9( ) 10( ) |
| It’s a fatal animal for humans | 0( ) 1( ) 2( ) 3( ) 4( ) 5( ) 6( ) 7( ) 8( ) 9( ) 10( ) |
| It is a completely harmless animal | 0( ) 1( ) 2( ) 3( ) 4( ) 5( ) 6( ) 7( ) 8( ) 9( ) 10( ) |
| It usually ignores humans | 0( ) 1( ) 2( ) 3( ) 4( ) 5( ) 6( ) 7( ) 8( ) 9( ) 10( ) |
| It tends to attack humans | 0( ) 1( ) 2( ) 3( ) 4( ) 5( ) 6( ) 7( ) 8( ) 9( ) 10( ) |
| It usually runs away from humans | 0( ) 1( ) 2( ) 3( ) 4( ) 5( ) 6( ) 7( ) 8( ) 9( ) 10( ) |
| It is important for nature | 0( ) 1( ) 2( ) 3( ) 4( ) 5( ) 6( ) 7( ) 8( ) 9( ) 10( ) |
|  |  |
| **ATTITUDINAL REPRESENTATIONS** | **SCALES** |
| I like the animal | 0( ) 1( ) 2( ) 3( ) 4( ) 5( ) 6( ) 7( ) 8( ) 9( ) 10( ) |
| I think the animal is ugly | 0( ) 1( ) 2( ) 3( ) 4( ) 5( ) 6( ) 7( ) 8( ) 9( ) 10( ) |
| I don’t go close to it | 0( ) 1( ) 2( ) 3( ) 4( ) 5( ) 6( ) 7( ) 8( ) 9( ) 10( ) |
| I like being close to this animal | 0( ) 1( ) 2( ) 3( ) 4( ) 5( ) 6( ) 7( ) 8( ) 9( ) 10( ) |
| I don’t like the noise the animal makes | 0( ) 1( ) 2( ) 3( ) 4( ) 5( ) 6( ) 7( ) 8( ) 9( ) 10( ) |
| I’m afraid of the animal | 0( ) 1( ) 2( ) 3( ) 4( ) 5( ) 6( ) 7( ) 8( ) 9( ) 10( ) |
| I can’t stand this animal | 0( ) 1( ) 2( ) 3( ) 4( ) 5( ) 6( ) 7( ) 8( ) 9( ) 10( ) |
| The animal gives me nightmares | 0( ) 1( ) 2( ) 3( ) 4( ) 5( ) 6( ) 7( ) 8( ) 9( ) 10( ) |
| The animal should be extinct | 0( ) 1( ) 2( ) 3( ) 4( ) 5( ) 6( ) 7( ) 8( ) 9( ) 10( ) |
| I don’t care if the animal lives in my house/property | 0( ) 1( ) 2( ) 3( ) 4( ) 5( ) 6( ) 7( ) 8( ) 9( ) 10( ) |
| If there was a population of this animal in my yard or property I would take steps to eliminate it. | 0( ) 1( ) 2( ) 3( ) 4( ) 5( ) 6( ) 7( ) 8( ) 9( ) 10( ) |
| I agree that this animal is protected by law | 0( ) 1( ) 2( ) 3( ) 4( ) 5( ) 6( ) 7( ) 8( ) 9( ) 10( ) |
| I usually kill it when I find it or ask someone for help to kill it. | 0( ) 1( ) 2( ) 3( ) 4( ) 5( ) 6( ) 7( ) 8( ) 9( ) 10( ) |

4. Check one or more of the options below corresponding to the medium where you heard most about or had access to information about the animal presented:

( ) Where you live (house, family, friends); ( ) School; ( ) Media in general (TV, internet, films); Others:_________________________________

5. Does this animal have any importance for you? ( ) Yes ( ) No. Why? _____________________________________________________________________


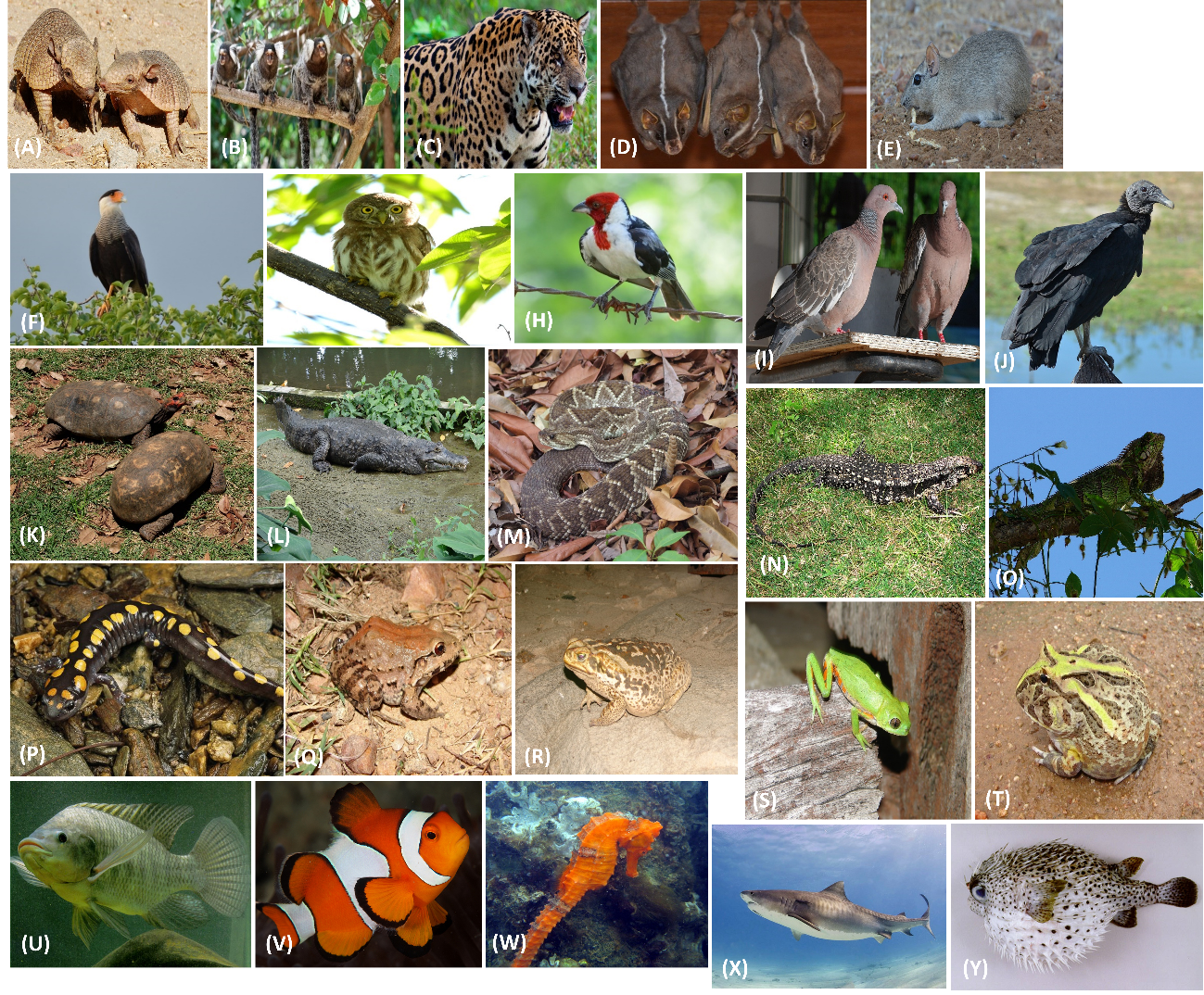
**Figure 1.** Species projected to students in the study: (A) six-banded armadillo (*Euphractus* *sexcinctus*); (B)marmoset *(Callithrix jacchus*); (C) jaguar (*Panthera onca*); (D) great fruit-eating bat (*Artibeus lituratus*); (E) Brazilian guinea pig (*Cavia aperea*); (F) crested carcara (*Caracara plancus*); (G) Ferruginous pygmy owl (*Glaucidium brasilianum*); (H) red-cowled cardinal (*Paroaria dominicana*); (I) Picazuro pigeon (*Patagioenas picazuro*); (J) black vulture (*Coragyps atratus*); (K) Red-footed tortoise (*Chelonoidis carbonaria*); (L) spectacled caiman/alligator (*Caiman crocodilus*); (M0 rattlesnake (*Crotalus durissus*); (N) Black and white tegu (*Salvator merianae*); (O) green iguana (*Iguana iguana*); (P) spotted salamander (*Ambystoma maculatum*); (Q) Northeastern pepper frog (*Leptodactylus vastus*); (R) Jimi toad (*Rhinella jimi*); (S) tree frog (*Phyllomedusa nordestina*); (T) horned frog (*Ceratophrys joazeirensis*); (U) trahira (*Hoplias malabaricus*); (V) clownfish (*Amphiprion ocellaris*); (W) longsnout seahorse (*Hippocampus reidi*); (X) tiger shark (*Galeocerdo cuvier*); (Y) and spot-fin porcupinefish (*Diodon hystrix*). Photo credits: (A), (B), (D), (E), (F), (G), (H), (I), (J) John Philip Medcraft; (C) Hugo Fernandes Ferreira; (K), (M), (N), (O), (Q), (R), (S), (T) Washington Vieira; (L) Rômulo Alves; (P) George Grall; (U) Olaya-Nieto C.W. (FishBase); (V) Redfinasia.com; (W) Renato; (X) Albert Kok; (Y) Hermosa Jr. G.V. (Fishbase).
